# Associations of physical activity, sedentary time, and cardiorespiratory fitness with heart rate variability in 6- to 9-year-old children: the PANIC study

**DOI:** 10.1007/s00421-019-04231-5

**Published:** 2019-09-18

**Authors:** Aapo Veijalainen, Eero A. Haapala, Juuso Väistö, Marja H. Leppänen, Niina Lintu, Tuomo Tompuri, Santeri Seppälä, Ulf Ekelund, Mika P. Tarvainen, Kate Westgate, Søren Brage, Timo A. Lakka

**Affiliations:** 1 Institute of Biomedicine, School of Medicine, University of Eastern Finland, Kuopio Campus, PO Box 1627, 70211 Kuopio, Finland; 2 Faculty of Sport and Health Sciences, University of Jyväskylä, Jyväskylä, Finland; 3 Folkhälsan Research Center, Helsinki, Finland; 4 Department of Sport Medicine, Norwegian School of Sport Sciences, Oslo, Norway; 5 Department of Clinical Physiology and Nuclear Medicine, Kuopio University Hospital, Kuopio, Finland; 6 Department of Applied Physics, University of Eastern Finland, Kuopio, Finland; 7 MRC Epidemiology Unit, Institute of Metabolic Science, School of Clinical Medicine, University of Cambridge, Cambridge, UK; 8 Kuopio Research Institute of Exercise Medicine, Kuopio, Finland

**Keywords:** Physical activity, Cardiorespiratory fitness, Autonomic nervous system, Children

## Abstract

**Purpose:**

To study the associations of physical activity (PA), sedentary time (ST), and cardiorespiratory fitness (CRF) with heart rate variability (HRV) in children.

**Methods:**

The participants were a population sample of 377 children aged 6–9 years (49% boys). ST, light PA (LPA), moderate PA (MPA), vigorous PA (VPA), and moderate-to-vigorous PA (MVPA), and PA energy expenditure (PAEE) were assessed using a combined heart rate and movement sensor, maximal power output per kilograms of lean body mass as a measure of CRF by maximal cycle ergometer exercise test, and HRV variables (SDNN, RMSSD, LF, and HF) using 5 min resting electrocardiography. Data were analysed by linear regression adjusted for years from peak height velocity.

**Results:**

In boys, ST was inversely associated (*β* = − 0.185 to − 0.146, *p* ≤ 0.049) and MVPA, VPA, PAEE, and CRF were directly associated (*β* = 0.147 to 0.320, *p* ≤ 0.048) with HRV variables. CRF was directly associated with all HRV variables and PAEE was directly associated with RMSSD after mutual adjustment for ST, PAEE, and CRF (*β* = 0.169 to 0.270, *p* ≤ 0.046). In girls, ST was inversely associated (*β* = − 0.382 to − 0.294, *p* < 0.001) and LPA, MPA, VPA, MVPA, and PAEE were directly associated with HRV variables (*β* = 0.144 to 0.348, *p* ≤ 0.049). After mutual adjustment for ST, PAEE, and CRF, only the inverse associations of ST with HRV variables remained statistically significant.

**Conclusions:**

Higher ST and lower PA and CRF were associated with poorer cardiac autonomic nervous system function in children. Lower CRF in boys and higher ST in girls were the strongest correlates of poorer cardiac autonomic function.

**Electronic supplementary material:**

The online version of this article (10.1007/s00421-019-04231-5) contains supplementary material, which is available to authorized users.

## Introduction

Globally, cardiovascular diseases cause remarkable individual, public health, and economic burden (Leal et al. 2006; Bloom et al. 2011; Laslett et al. 2012). Although cardiovascular diseases are typically affecting adults, these diseases have their origin in childhood (Celermajer and Ayer 2006). Therefore, the prevention of cardiovascular diseases, including the promotion of a physically active lifestyle, should start in childhood (Kavey et al. 2003; Celermajer and Ayer 2006).

The beneficial effects of physical activity (PA) on risk factors for cardiovascular diseases and their clinical manifestations in adults have been extensively documented (Blair and Morris 2009). Recent evidence suggests that increased PA and decreased sedentary time (ST) improve traditional cardiometabolic risk factors, such as insulin resistance and dyslipidemia, already among children (Väistö et al. 2019). However, the reduced incidence of cardiovascular diseases is only partly attributed to reduced levels of traditional risk factors due to increased PA and the mechanisms through which PA reduces cardiovascular risk are not completely understood (Green et al. 2008).

One of the mechanisms for the association between increased PA and reduced risk of cardiovascular diseases may be alterations in cardiac autonomic nervous system function, which can be assessed by heart rate variability (HRV) (Joyner and Green 2009). Decreased HRV has been associated with multiple cardiovascular diseases, risk factors, mortality and other clinical conditions in adults, but the studies in children are still limited (Villareal et al. 2002; Seppälä et al. 2014). A recent systematic literature review concluded that higher levels of moderate-to-vigorous PA (MVPA) were associated with better cardiac autonomic nervous system function in children and adolescents (Oliveira et al. 2017). However, the literature review also highlighted that the evidence on the association between PA and cardiac autonomic nervous system function is still limited and called for further studies investigating the associations of the whole spectrum of PA intensity and the confounding factors and physiological mechanisms for the association, such as age, sex, biological maturation, and body weight (Oliveira et al. 2017). Furthermore, few studies with inconsistent results have investigated the association between cardiorespiratory fitness (CRF) and HRV in children (Oliveira et al. 2017).

The purpose of our study was to investigate the independent and joint associations of different PA intensities, ST, and CRF with cardiac autonomic nervous system function, assessed by HRV, in children aged 6–9 years controlling comprehensively for possible confounding factors.

## Methods

### Study design and study population

The present cross-sectional analyses are based on the baseline data of the physical activity and nutrition in children (PANIC) study, which is a physical activity and dietary intervention and follow-up study in a population sample of children from the city of Kuopio, Finland. Altogether 736 children aged 6–9 years from primary schools of Kuopio were invited to participate in the baseline examination in 2007–2009. A total of 512 children (70% of those invited) participated in the baseline examinations. Six children were excluded from the study at baseline because of physical disabilities that could hamper participation in the intervention or had limited time or motivation to participate in the study. The participants did not differ in sex distribution, age, or body mass index standard deviation score from all children who started the first grade in Kuopio in 2007–2009 based on data from the standard school health examinations performed for all Finnish children before the first grade (data not shown). In the present analyses, valid data for HRV, PA, ST, and CRF were available for 377 children. The study protocol was approved by the Research Ethics Committee of the Hospital District of Northern Savo, Kuopio, Finland. All participating children and their parents provided written informed consent.

### Study protocol

The children and their parents or caregivers arrived at the exercise and health laboratory at the Institute of Biomedicine at 08.00 am or 09.15 am after fasting for at least 12 h. The children were pre-informed to abstain from caffeinated drinks for at least 12 h and avoid strenuous PA for at least 24 h before the visit but continue using their medications. A physician performed a clinical examination before the maximal exercise test and the HRV assessments. The children were also offered a breakfast and were asked to rest to standardize the conditions before the HRV assessments and the maximal exercise test that were performed about an hour after having the breakfast. The visit was rescheduled for children who had suffered from an illness or a condition that could hamper biochemical analyses performed using blood samples, cause a risk during the exercise test, or make it difficult to perform the exercise test. The measurements were performed at a temperature of 21 ± 1 °C and at a humidity of 30 ± 10%.

### Assessment of heart rate variation

The assessment of HRV has been explained in more detail elsewhere (Seppälä et al. 2014). Briefly, electrocardiography was registered for five minutes after a 10-minute rest in a supine position and before the exercise test according to the conventional 12-lead system using the Cardiosoft^®^ V6.5 Diagnostic System (GE Healthcare Medical Systems, Freiburg, Germany) at a frequency of 500 Hz. The electrocardiographic data were analysed using the Kubios^®^ HRV software (Kubios Ltd., Kuopio, Finland), and the details of the techniques and analysis methods employed to assess HRV have been described elsewhere (Tarvainen et al. 2014). Briefly, the R-wave peaks were first detected using an adaptive QRS detection algorithm, and the RR interval time series (time intervals between successive R waves as a function of R-wave time instants) were formed. Prior to the analysis, the data were checked for potential ectopic or aberrant beats and, if necessary, such erroneous beats were corrected using interpolation methods. The HRV variables used in the analyses included the standard deviation of all RR intervals (SDNN), a marker of overall HRV, and the root mean square of successive RR interval differences (RMSSD), a marker for parasympathetic activity (Malliani et al. 1991). These HRV variables assess HRV in the time domain with a lower value indicating a lowered parasympathetic modulation. In addition, we assessed HRV in the frequency domain by calculating high-frequency power (HF 0.15–0.40 Hz), which represents the parasympathetic modulation, low-frequency power (LF 0.04–0.15 Hz), which represents a mixture of the sympathetic and parasympathetic modulation, and LF/HF, which estimates the balance between sympathetic and parasympathetic nervous system activity (Shaffer and Ginsberg 2017). For comparison purposes, we also calculated HRV variables adjusted for the mean of RR interval, as suggested elsewhere (Gąsior et al. 2018).

### Assessment of cardiorespiratory fitness

CRF was assessed by a maximal exercise test using the Ergoselect 200 K® electromagnetic bicycle ergometer with a paediatric saddle module (Ergoline, Bitz, Germany), as explained in more detail previously (Lintu et al. 2013; Tompuri et al. 2015a). The exercise test protocol included a 3 min warm-up period at 5 W, a 1 min steady-state period at 20 W, an exercise period with a workload increase of 1 W every 6 s until exhaustion, and a 4 min cooling-down period at 5 W. The children were asked to keep the cadence stable and within 70–80 revolutions per minute. Exhaustion was defined as the inability to maintain the cadence above 65 revolutions per minute regardless of vigorous verbal exhortation. The exercise test was considered maximal if the reason for terminating the test indicated maximal effort and maximal cardiorespiratory capacity and if the child could not maintain the cadence above 65 revolutions per minute regardless of vigorous verbal exhortation and reached at least 90% of maximal predicted heart rate (Karila et al. 2001; Tompuri et al. 2015a). Maximal workload expressed in watts per kilograms of lean body mass was used as a measure of CRF.

### Assessment of physical activity and sedentary time

PA and ST were assessed using a combined heart rate and movement sensor Actiheart® (CamNtech Ltd., Papworth, UK) for a minimum of four consecutive days without interruption, including two weekdays and two weekend days, analysed in 60 s epochs (Brage et al. 2005). The combined heart rate and movement sensor were attached to the child’s chest with two standard electrocardiographic electrodes (Bio Protech Inc., Wonju, South Korea). The children were asked to wear the monitor continuously including sleep and water-based activities and not to change their usual behaviour during the monitoring period. Heart rate data were cleaned (Stegle et al. 2008) and individually calibrated using parameters obtained from the maximal cycle exercise test (Brage et al. 2007) and were combined with movement sensor data to derive PA energy expenditure (PAEE). Instantaneous PAEE, i.e. PA intensity, was estimated using branched equation modelling (Brage et al. 2004) and was expressed as time spent at intensity levels of standard metabolic equivalents (METs), one MET corresponding to 71.2 J/min/kg, in minutes per day. Non-wear time was taken into account during summation to minimise potential diurnal bias caused by imbalances in wear time. For these analyses, time spent in intensity categories was summarized as sedentary time (ST) (≤ 1.50 METs), light PA (LPA 1.51–4.00 METs), moderate PA (MPA 4.01–7.00 METs, and vigorous PA (> 7.00 METs). MVPA included MPA and VPA. These broader MET thresholds have commonly been applied in studies on PA among children and youth (Janssen and Leblanc 2010). The average sleep duration, that was inferred from the combined heart rate and movement data by a trained exercise specialist and was confirmed by a physician, was subtracted from ST to obtain the final ST for the analyses (Collings et al. 2017). The time of falling asleep was defined as accelerometer counts decreasing to zero and heart rate to a plateau level. The time of waking up was defined as accelerometer counts increasing and remaining above zero and heart rate increasing and remaining above the plateau level. We accepted PA and ST data for the statistical analyses if there was a minimum of 48 h of activity recording in weekday and weekend day hours that included at least 12 h from morning (3–9 am), noon (9 am–3 pm), afternoon (3–9 pm), and night (9 pm–3 am) to avoid potential bias from over-representing specific times and activities of the days.

### Assessment of cardiometabolic risk factors

Body weight was measured twice after overnight fasting, bladder emptied, in standing position, and in light underwear using a calibrated InBody® 720 bioelectrical impedance device (Biospace, Seoul, South Korea) to accuracy of 0.1 kilograms. The mean of these two values was used in the analyses. Body height was measured three times in the Frankfurt plane without shoes by a wall-mounted stadiometer to accuracy of 0.1 cm. The mean of two nearest values was used in the analyses. Body mass index was calculated as body weight in kilograms divided by body height in meters squared. Body mass index standard deviation score was computed by the national references (Saari et al. 2011). Overweight was defined using the age- and sex-specific cutoffs of body mass index according to the International Obesity Task Force criteria (Cole et al. 2000). Waist circumference was measured three times at the end of expiration at mid-distance between the bottom of the rib cage and the top of the iliac crest with a non-stretchable measuring tape to accuracy of 0.1 cm. The mean of two nearest values was used in the analyses. Body fat percentage (BF%) and lean body mass were measured bladder emptied, in supine position, in light clothing and after removing all metal objects using the Lunar Prodigy Advance® dual-energy X-ray absorptiometry device (GE Medical Systems, Madison, Wisconsin, USA) (Tompuri et al. 2015b).

A research nurse took blood samples in the morning after a 12 h overnight fast. Plasma glucose was measured by a hexokinase method, serum insulin by an electrochemiluminescence immunoassay, plasma triglycerides by a colorimetric enzymatic assay, and plasma high-density lipoprotein cholesterol by a homogeneous colorimetric enzymatic assay (Viitasalo et al. 2014).

A research nurse measured systolic and diastolic blood pressure from the right arm using the Heine Gamma® G7 aneroid sphygmomanometer (Heine Optotechnik, Herrsching, Germany) to accuracy of 2 mmHg. The measurement protocol included a 5 min seated resting period followed by three measurements with a 2 min interval in between. The average of all three values of systolic blood pressure and the average of all three values of diastolic blood pressure were used in the analysis.

Age-, sex-, and height-standardized *z*-scores were calculated for waist circumference, insulin, glucose, triglycerides, reciprocal of high-density lipoprotein cholesterol, and the mean of systolic and diastolic blood pressure. Thereafter, cardiometabolic risk score (CRS) was calculated as the sum of those z scores: a larger score indicating a higher cardiometabolic risk (Viitasalo et al. 2014).

### Assessment of maturation

The research physician assessed pubertal status using the five-stage scale described by Tanner (Marshall and Tanner 1969, 1970). The boys were defined as having entered clinical puberty if their testicular volume assessed by an orchidometer was ≥ 4 mL (stage ≥ 2). The girls were defined as having entered clinical puberty if their breast development had started (stage ≥ 2). As almost all children were prepubertal (98%), years from peak height velocity (PHV) were used as an indicator of maturity (Malina et al. 2015), and it was calculated separately for boys and girls using formulas provided by Moore (Moore et al. 2015).

## Statistical methods

All statistical analyses were performed using the SPSS statistical software, version 24.0 (IBM Corp., Armonk, NY, USA). The analyses were performed separately for boys and girls, because we found that sex modified the associations of ST, LPA, and MPA with HRV variables. Basic characteristics were presented as arithmetic means (standard deviations, SD) or frequencies (percentages, %) and were compared between boys and girls using the Student’s *t *test, the Mann–Whitney *U *test, or the Chi Square test. Before the analyses, the HRV variables (SDNN, RMSSD, HF, LF, and LF/HF ratio) were logarithmically transformed due to their skewed distributions. We used the linear regression analysis to investigate the associations of ST, LPA, MPA, VPA, MVPA, and CRF with the HRV variables. First, ST, LPA, MPA, VPA, MVPA, and CRF were forced one by one with years from PHV into the linear regression models, and then BF% or CRS was additionally forced into the models. Second, ST, PAEE, and CRF were forced simultaneously into the linear regression model with years from PHV, BF%, and CRS. The linear regression analyses were also repeated using HRV variables adjusted for the mean of RR interval (SDNN_adjusted_, RMSSD_adjusted_, HF_adjusted_, LF_adjusted_, and LF/HF ratio_adjusted_). Moreover, we studied differences in the HRV variables between children with low (≤ sex-specific median) and high (> sex-specific median) PAEE and CRF and between children with low (≤ sex-specific median) and high (> sex-specific median) ST and CRF using general linear models adjusted for years from PHV, BF%, and CRS. There was no considerable multicollinearity between variables used in the statistical analyses, because the Variance Inflation Factor in all analyses was under 3.4. Residuals in all models were normal. Associations with a *p *value of < 0.05 were considered statistically significant.

## Results

### Basic characteristics

Children in this study were slightly older (7.65 years versus 7.56 years, *p* = 0.042) than the 512 children in the baseline study group but did not differ in height, weight, waist circumference, systolic blood pressure, or diastolic blood pressure from the 135 children who were excluded. Almost all children (98%) were prepubertal. Boys were taller and heavier, had lower BF%, and were further away from PHV than girls (Table 1). Boys also had a higher waist circumference, lower insulin, higher glucose, and lower triglycerides than girls (Table 1). Boys had a lower heart rate and higher mean of RR intervals than girls. Boys also accumulated less LPA and more MPA, VPA, and MVPA and had a higher PAEE and CRF than girls (Table 1).

**Table 1:** Characteristics of children

	Boys (*n* = 185)		Girls (*n* = 192)		*P *value for sex difference^c^
	Mean	SD	Mean	SD	
Age (years)	7.7	0.4	7.6	0.4	0.149
Peak height velocity (years)	− 4.4	0.3	− 3.6	0.3	** < 0.001**
Body height (cm)	130	5.3	128	5.6	** < 0.001**
Body weight (kg)	27.5	5.0	26.2	4.8	**0.003**
Body mass index standard deviation score^a^ (kg/m^2^)	− 0.2	1.1	− 0.2	1.1	0.61
Body fat content (%)	17.4	8.0	21.9	7.5	** < 0.001**
Overweight or obese^b^ (N, %)	22 (11.9)	24 (12.5)	0.857		
Waist circumference (cm)	57.5	5.9	55.6	5.7	** < 0.001**
Insulin (mU/L)	4.2	2.6	4.7	2.4	**0.008**
Glucose (mmol/L)	4.9	0.3	4.7	0.4	** < 0.001**
Triglycerides (mmol/L)	0.58	0.25	0.62	0.25	**0.034**
High-density lipoprotein cholesterol (mmol/L)	1.63	0.31	1.58	0.29	0.084
Systolic blood pressure (mm Hg)	100.3	7.1	99.6	7.2	0.358
Diastolic blood pressure (mm Hg)	62.1	6.8	61.1	7.5	0.233
Cardiometabolic risk score	0.0	3.7	− 0.3	3.3	0.475
*Heart rate variability variables*					
Mean heart rate (beats/min)	83	10.0	85	9.7	**0.043**
Mean of RR intervals (ms)	739	89	720	85	**0.034**
Standard deviation of all RR intervals (ms)	62.0	28.8	58.5	27.9	0.291
Root mean square of successive RR interval differences (ms)	70.2	43.2	65.5	39.3	0.537
Low-frequency power (ms^2^)	1309	985	1134	961	0.050
High-frequency power (ms^2^)	53.1	16.9	55.3	15.7	0.313
Ratio of low- and high-frequency power	1.0	1.0	0.8	0.6	0.148
*Sedentary time, physical activity, and cardiorespiratory fitness*					
Sleep time (hours/night)	9.6	0.5	9.7	0.5	0.444
Sedentary time (min/day)	231	131	242	130	0.390
Light physical activity (min/day)	496	102	520	109	**0.011**
Moderate physical activity (min/day)	103	56	82	47	** < 0.001**
Vigorous physical activity (min/day)	30	26	16	16	** < 0.001**
Moderate-to-vigorous physical activity (min/day)	133	67	97	55	** < 0.001**
Physical activity energy expenditure (kJ/kg/day)	104	34	90	28	** < 0.001**
Cardiorespiratory fitness (Watt/kg)	3.8	0.5	3.6	0.5	** < 0.001**

*P *values < 0.05 indicating statistically significant differences are bolded

*SD* standard deviation

^a^According to Saari et al. (2011)

^b^According to Cole et al. (2000)

^c^*t *test or Mann–Whitney *U *test for continuous variables and Chi-square test for being overweight or obese

### Associations of ST, PA, and CRF with HRV variables

In boys, ST was inversely associated with SDNN, RMSSD, LF, and HF after adjustment for years from PHV (Table 2). These associations were no longer statistically significant after further adjustment for BF% or CRS. MPA was directly associated with RMSSD after adjustment for years from PHV (Table 2). MVPA, VPA, PAEE, and CRF were directly associated with SDNN, RMSSD, LF, and HF and inversely associated with LF/HF ratio adjusted for years from PHV (Table 2). The association of MPA with RMSSD and the associations of MVPA and PAEE with LF/HF ratio were no longer statistically significant after further adjustment for BF% or CRS. CRF was directly associated with SDNN, RMSSD, LF, and HF and inversely associated with LF/HF ratio after adjusted for ST, PAEE, years from PHV, BF%, and CRS (Table 4). PAEE was directly associated with RMSSD adjusted for ST, CRF, years from PHV, BF%, and CRS (Table 4).

**Table 2:** Individual associations of sedentary time, physical activity, and cardiorespiratory fitness with heart rate variability variables in boys

	SDNN			RMSSD			LF			HF			LF/HF		
	*β*	95% CI	*p*	*β*	95% CI	*p*	*β*	95% CI	*p*	*β*	95% CI	*p*	*β*	95% CI	*p*
ST	**− 0.166**	**− 0.31|− 0.02**	**0.026**	**− 0.185**	**− 0.33|− 0.04**	**0.012**	**− 0.156**	**− 0.30|− 0.01**	**0.037**	**− 0.146**	**− 0.29|− 0.001**	**0.049**	0.060	**− **0.09|0.21	0.417
LPA	0.031	− 0.12|0.18	0.682	0.021	**− 0**.13|0.18	0.773	0.069	**− **0.08|0.22	0.355	0.006	**− **0.14|0.15	0.936	0.070	**− **0.08|0.22	0.343
MPA	0.113	**− **0.03|0.26	0.127	**0.158**	**0.01|0.30**	**0.032**	0.067	**− **0.08|0.21	0.369	0.111	**− **0.03|0.26	0.133	**− **0.105	**− **0.25|0.04	0.154
MVPA	**0.208**	**− 06|0.35**	**0.005**	**0.252**	**0.11|0.39**	**0.001**	**0.147**	**0.001|0.29**	**0.048**	**0.210**	**0.07|0.35**	**0.004**	**− 0.175**	**− 0.32|− 0.03**	**0.017**
VPA	**0.284**	**0.15|0.42**	** < 0.001**	**0.301**	**0.16|0.44**	** < 0.001**	**0.229**	**0.09|0.37**	**0.002**	**0.294**	**0.16|0.43**	** < 0.001**	**− 0.219**	**− 0.36|− 0.08**	**0.002**
PAEE	**0.246**	**0.10|0.39**	**0.001**	**0.283**	**0.14|0.43**	** < 0.001**	**0.201**	**0.06|0.35**	**0.007**	**0.240**	**0.10|0.38**	**0.001**	**− 0.163**	**− 0.31|− 0.02**	**0.027**
CRF	**0.279**	**0.14|0.42**	** < 0.001**	**0.320**	**0.18|0.46**	** < 0.001**	**0.214**	**0.07|0.36**	**0.004**	**0.278**	**0.14|0.42**	** < 0.001**	**− 0.209**	**− 0.35|− 0.07**	**0.005**

Values are standardized regression coefficients (*β*), 95% confidence intervals (CI), and *p *values from linear regression analyses in which each ST, PA, and CRF variable was entered individually with years from peak height velocity into the models. *P *values < 0.05 indicating statistically significant associations are in bold

*ST* sedentary time, *PA* physical activity, *CRF* cardiorespiratory fitness, *SDNN* standard deviation of all RR intervals, *RMSSD* root mean square of successive RR interval differences, *LF* low-frequency power, *HF* high-frequency power, *LPA* light PA, *MPA* moderate PA, *VPA* vigorous PA, *MVPA* moderate-to-vigorous PA, *PAEE* physical activity energy expenditure

In girls, ST was inversely associated with SDNN, RMSSD, LF, and HF and directly associated with LF/HF ratio after adjustment for years from PHV (Table 3). LPA, MPA, MVPA, VPA, and PAEE were directly associated with SDNN, RMSSD, LF, and HF and inversely associated with LF/HF ratio adjusted for years from PHV (Table 3). CRF was inversely associated with LF/HF ratio after adjustment for years from PHV (Table 3). Further adjustment for BF% or CRS had no effect on these associations, except that the inverse association with LPA and LF/HF ratio was no longer significant. Only the inverse associations of ST with SDNN, RMSSD, LF, and HF remained statistically significant after adjustment for PAEE, CRF, years from PHV, BF%, and CRS (Table 4).

**Table 3:** Individual associations of sedentary time, physical activity, and cardiorespiratory fitness with heart rate variability variables in girls

	SDNN			RMSSD			LF			HF			LF/HF		
	*β*	95% CI	*p*	*β*	95% CI	*p*	*β*	95% CI	*p*	*β*	95% CI	*p*	*β*	95% CI	*p*
ST	**− 0.370**	**− 0.50|− 0.24**	** < 0.001**	**− 0.382**	**− 0.52|− 0.25**	** < 0.001**	**− 0.294**	**− 0.43|− 0.16**	** < 0.001**	**− 0.363**	**− 0.50|− 0.23**	** < 0.001**	**0.244**	**0.10|0.38**	**0.001**
LPA	**0.292**	**0.16|0.43**	** < 0.001**	**0.296**	**0.16|0.43**	** < 0.001**	**0.245**	**0.11|0.39**	**0.001**	**0.271**	**0.13|0.41**	** < 0.001**	**− 0.152**	**− 0.29|− 0.01**	**0.036**
MPA	**0.210**	**0.070|0.35**	**0.004**	**0.238**	**0.10|0.38**	**0.001**	**0.144**	**0.001|0.29**	**0.049**	**0.218**	**0.08|0.36**	**0.003**	**− 0.186**	**− 0.33|− 0.04**	**0.011**
MVPA	**0.274**	**0.14|0.41**	** < 0.001**	**0.301**	**0.16|0.44**	** < 0.001**	**0.182**	**0.04|0.32**	**0.012**	**0.284**	**0.15|0.42**	** < 0.001**	**− 0.249**	**− 0.39|− 0.11**	**0.001**
VPA	**0.332**	**0.20|0.47**	** < 0.001**	**0.346**	**0.21|0.48**	** < 0.001**	**0.210**	**0.07|0.35**	**0.003**	**0.348**	**0.21|0.48**	** < 0.001**	**− 0.320**	**− 0.46|− 0.18**	** < 0.001**
PAEE	**0.343**	**0.21|0.48**	** < 0.001**	**0.371**	**0.24|0.51**	** < 0.001**	**0.240**	**0.10|0.38**	**0.001**	**0.347**	**0.21|0.48**	** < 0.001**	**− 0.282**	**− 0.42|− 0.13**	** < 0.001**
CRF	0.080	− 0.07|0.23	0.277	0.132	− 0.01|0.28	0.073	− 0.001	− 0.15|0.15	0.985	0.119	− 0.03|0.26	0.108	**− 0.195**	**− 0.34|− 0.05**	**0.008**

Values are standardized regression coefficients (*β*), 95% confidence intervals (CI), and *p *values from linear regression analyses in which each ST, PA, and CRF variable was entered individually with years from peak height velocity into the models. *P *values < 0.05 indicating statistically significant associations are in bold

*ST* sedentary time, *PA* physical activity, *CRF* cardiorespiratory fitness, *SDNN* standard deviation of all RR intervals, *RMSSD* root mean square of successive RR interval differences, *LF* low-frequency power, *HF* high-frequency power, *LPA* light PA, *MPA* moderate PA, *VPA* vigorous PA, *MVPA* moderate-to-vigorous PA, *PAEE* physical activity energy expenditure

**Table 4:** Mutually adjusted associations of sedentary time, physical activity, and cardiorespiratory fitness with heart rate variability variables

	SDNN			RMSSD			LF			HF			LF/HF		
	*β*	95% CI	*p*	*β*	95% CI	*p*	*β*	95% CI	*p*	*β*	95% CI	*p*	*β*	95% CI	*p*
*Boys*															
ST	0.088	− 0.16|0.34	0.474	0.117	− 0.12|0.36	0.333	0.005	− 0.25|0.26	0.966	0.142	− 0.10|0.39	0.248	− 0.227	− 0.48|0.02	0.066
PAEE	0.220	− 0.04|0.49	0.097	**0.259**	**0.004|0.52**	**0.046**	0.153	− 0.12|0.43	0.261	0.232	− 0.03|0.50	0.078	− 0.204	− 0.47|0.06	0.124
CRF	**0.231**	**0.08|0.38**	**0.004**	**0.270**	**0.12|0.42**	**0.001**	**0.169**	**0.010|0.33**	**0.038**	**0.232**	**0.08|0.38**	**0.003**	**− 0.184**	**− 0.34|− 0.03**	**0.020**
*Girls*															
ST	**− 0.301**	**− 0.56|− 0.06**	**0.015**	**− 0.279**	**− 0.53|− 0.04**	**0.022**	**− 0.338**	**− 0.60|− 0.09**	**0.008**	**− 0.269**	**− 0.53|− 0.03**	**0.031**	0.041	− 0.21|0.30	0.743
PAEE	0.101	− 0.15|0.36	0.420	0.120	− 0.13|0.37	0.334	0.011	− 0.25|0.27	0.930	0.112	− 0.14|0.37	0.376	− 0.170	− 0.43|0.09	0.190
CRF	0.035	− 0.12|0.19	0.646	0.084	− 0.07|0.23	0.267	− 0.018	− 0.17|0.14	0.815	0.072	− 0.08|0.22	0.348	− 0.140	− 0.29|0.02	0.077

Values are standardized regression coefficients (*β*), 95% confidence intervals (CI), and *p* values from linear regression analyses in which ST, PAEE, and CRF were entered simultaneously with years from peak height velocity, body fat percentage, and cardiometabolic risk score into the models. *P* values < 0.05 indicating statistically significant associations are in bold

*ST* sedentary time, *PA* physical activity, *CRF* cardiorespiratory fitness, *SDNN* standard deviation of all RR intervals, *RMSSD* root mean square of successive RR interval differences, *LF* low-frequency power, *HF* high-frequency power, *PAEE* PA energy expenditure

In boys, only VPA and CRF were directly associated with RMSSD_adjusted_ and LF_adjusted_ and inversely associated with LF/HF_adjusted_ after adjustment for years from PHV (Online resource 1). CRF was directly associated with RMSSD_adjusted_ and inversely with LF/HF ratio_adjusted_ after adjustments for ST, PAEE, years from PHV, BF%, and CRS (Online resource 2).

In girls, ST was inversely associated with SDNN _adjusted_, RMSSD _adjusted_, and HF and directly associated with LF/HF ratio_adjusted_ after adjustment for years from PHV (Online resource 1). LPA was directly associated with SDNN_adjusted_, RMSSD_adjusted_, and HF after adjustment for years from PHV (Online resource 1). MPA was inversely associated with LF/HF ratio_adjusted_ after adjustment for years from PHV (Online resource 1). MVPA was directly associated with RMSSD_adjusted_ and HF_adjusted_ and inversely with LF/HF ratio_adjusted_ after adjustment for years from PHV (Online resource 1). VPA and PAEE were directly associated with SDNN_adjusted_, RMSSD_adjusted_, and LF_adjusted_ and inversely associated with LF/HF ratio_adjusted_ after adjustment for years from PHV (Online resource 1). CRF was inversely associated with LF/HF ratio_adjusted_ after adjustment for years from PHV (Online resource 1). ST was inversely associated with SDNN_adjusted_, RMSSD_adjusted,_ LF_adjusted_, and HF_adjusted_ after adjustments for CRF, PAEE, years from PHV, BF%, and CRS (Online resource 2).

**Fig. 1:**
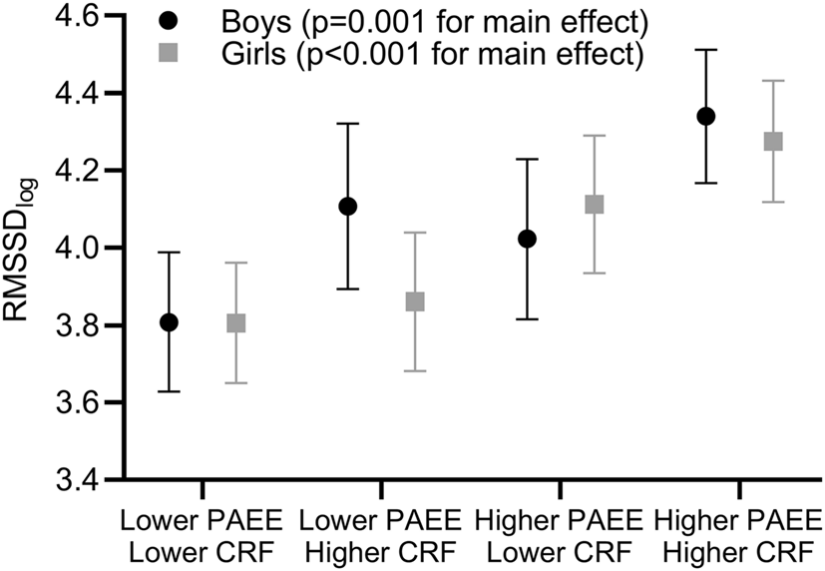
Means (95% confidence intervals) of root mean square of successive RR interval differences (RMSSD) among children with low (≤ sex-specific median) and high (> sex-specific median) maximal workload per lean body mass (CRF) and physical activity energy expenditure (PAEE)

Boys and girls with a combination of lower CRF and lower PAEE had the lowest RMSSD and boys and girls with a combination of higher CRF and higher PAEE had the highest RMSSD adjusted for years from PHV, BF%, and CRS (*p* for the main effect = 0.001 for boys and < 0.001 for girls, Fig. 1). These results were similar for SDNN (*p* = 0.001 for boys and *p* = 0.001 for girls), LF (*p* = 0.038 for boys and *p* = 0.010 for girls), and HF (*p* = 0.014 for boys and *p* < 0.001 for girls) adjusted for years from PHV, BF%, and CRS. Girls with a combination of lower CRF and lower PAEE had the highest LF/HF ratio and girls with a combination of higher CRF and higher PAEE had the lowest LF/HF ratio adjusted for years from PHV, BF%, and CRS (*p* for the main effect = 0.013).

**Fig. 2:**
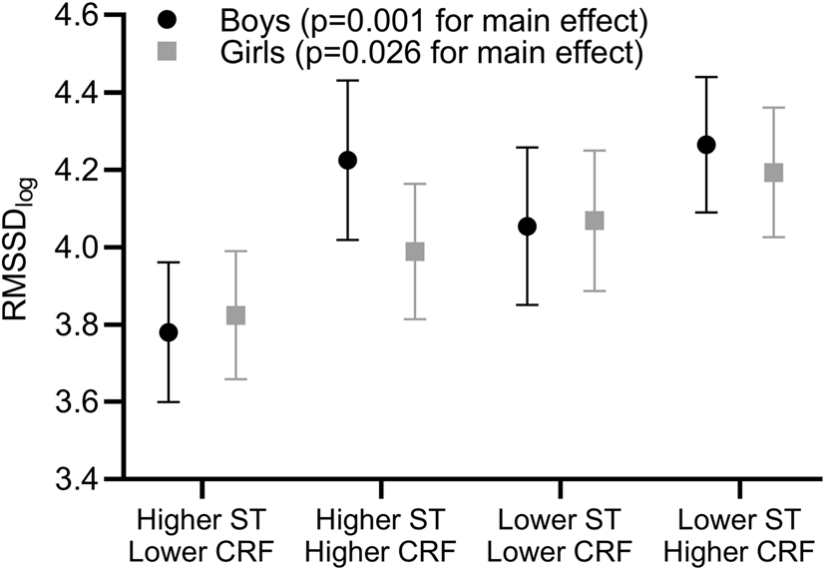
Means (95% confidence intervals) of root mean square of successive RR interval differences (RMSSD) among children with low (≤ sex-specific median) and high (> sex-specific median) maximal workload per lean body mass (CRF) and sedentary time (ST)

Boys and girls with a combination of higher ST and lower CRF had the lowest RMSSD and boys and girls with a combination of lower ST and higher CRF had the highest RMSSD after adjustment for years from PHV, BF%, and CRS (*p* = 0.001 for boys and *p* = 0.026 for girls, Fig. 2). These results were similar for SDNN (*p* = 0.034 for boys and *p* = 0.002 for girls), LF (*p* = 0.003 for boys), and HF (*p* = 0.009 for boys and *p* = 0.019 for girls) adjusted for years from PHV, BF%, and CRS.

## Discussion

### Main findings

The main finding of our study was that higher levels of PA, lower levels of ST, and higher CRF were associated with better cardiac autonomic nervous system function, as indicated by higher heart rate variability, in children aged 6–9 years independent of adiposity and the cluster of cardiometabolic risk factors. CRF was the strongest determinant of cardiac autonomic nervous system function in boys and ST was the strongest determinant of it in girls. Furthermore, we found the best cardiac autonomic nervous system function in boys and girls with a combination of higher PA and higher CRF or a combination of lower ST and higher CRF and the worst cardiac autonomic nervous system function in boys and girls with a combination of lower PA and lower CRF or a combination of higher ST and lower CRF. From a clinical point of view, the results of this study suggest that the beneficial effects of an active lifestyle go beyond traditional cardiovascular risk factors. Measuring HRV could thus help to target interventions.

### Associations of PA and ST with cardiac autonomic nervous system function

In line with previous evidence (Oliveira et al. 2017), we found that more time spent in PA was associated with better cardiac autonomic nervous system function independent of adiposity and the clustering of cardiometabolic risk factors. These associations were stronger in girls than in boys. Higher levels of LPA, MPA, and VPA were related to better cardiac autonomic nervous system function in girls, whereas the positive association between PA and cardiac autonomic nervous system function in boys was mainly attributed by the strong direct association between VPA and cardiac autonomic nervous system function. However, previous studies have mainly been carried out in adolescents. Moreover, PA has usually been assessed using uniaxial accelerometer (Gutin et al. 2005; Radtke et al. 2013), which tends to underestimate PA more than combined heart rate and motion sensors (Strath et al. 2001). Furthermore, HRV variables have been shown to differ among age groups and between sexes in children that highlights the need to study the determinants of HRV in different age groups of children and in boys and girls separately (Silvetti et al. 2001).

Krishnan and coworkers showed no association of PA with HRV in children aged 9 years, but they did not fully account for the confounding effect of pubertal stages (Krishnan et al. 2009). Michels and coworkers found a positive association between PA and HRV but they did not report whether the association was statistically significant (Michels et al. 2013). Moreover, we studied the associations of not only PA but also ST and CRF with cardiac autonomic nervous system function and found that ST was a more important correlate of HRV variables than PAEE in girls and that CRF was a more important correlate of HRV variables than PAEE in boys. The results of the present study in children provide additional evidence beyond the findings of previous studies among adolescents in that the positive association between PA and cardiac autonomic nervous system function exists already in mid-childhood and is independent of adiposity and cluster of cardiometabolic risk factors and maturation in girls but may be modified by ST in girls and by CRF in boys. These cross-sectional observations are also supported by the results of an exercise intervention study showing a beneficial effect of 4-month exercise training on parasympathetic activity in obese children aged 7–11 years (Gutin et al. 2000). Exercise training had no effect on body composition and CRF was assessed using a submaximal exercise test, so no conclusion could be made on the mediating effect of CRF or adiposity on the beneficial effects of exercise training on HRV. However, longitudinal follow-up studies and especially intervention studies in children are scarce, and further studies are needed to investigate whether PA improves cardiac autonomic nervous system function in children (da Silva et al. 2014).

To our knowledge, there is only one study on the association between ST and HRV in children where there was no such association in children aged 13 years (Oliveira et al. 2017,2018). However, higher ST has been related to lower HRV in adults (Buchheit et al. 2004; Hallman et al. 2015; Miyagi et al. 2019). Consistent with these findings in adults, we observed that higher ST was associated with poorer cardiac autonomic nervous system function in children. This association was stronger in girls than in boys, and it was independent of adiposity and the cluster of cardiometabolic risk factors.

One of the explanations for the observed associations of lower PA and higher ST with lower HRV could be that lower PA and higher ST decrease blood volume and left ventricular stroke volume that results in increased heart rate due to increased sympathetic activity (Hughson and Shoemaker 2015). The regulation of hemodynamics of the human body is complex and involves neural and humoral mechanisms (Haack and Zucker 2015; Besnier et al. 2017).

### Association of CRF with cardiac autonomic nervous system function

Some studies have found a positive association between CRF and cardiac autonomic nervous system function in children and adolescents, but the observations remain inconsistent (Oliveira et al. 2017, 2018). One explanation for the discrepancy may be that most of the previous studies have assessed CRF using indirect field tests or have used measures of CRF scaled by body mass (Oliveira et al. 2017) that could be confounded by adiposity and does not reflect physiological aerobic capacity (Ahn et al. 2013; Krachler et al. 2015; Tompuri et al. 2015a).

We observed that higher CRF was associated with better cardiac autonomic nervous system function independent of PA, ST, adiposity, and other cardiometabolic risk factors in children, particularly boys. In addition, CRF partly explained the positive associations of PA with HRV variables in boys suggesting that CRF mediates the relationship between PA and cardiac autonomic nervous system function.

The mechanisms underlying the association between CRF and cardiac autonomic nervous system function may be similar to those between PA and cardiac autonomic function, because especially more vigorous PA has been found to improve CRF (Ortega et al. 2008b). Although we showed a stronger association of PA with HRV in girls than in boys, the association of CRF with HRV was weaker in girls. The improvement of CRF by increasing PA has been observed to be smaller in girls than in boys that may be explained by differences in maturation and adiposity between sexes (Ortega et al. 2007, 2008b). However, genetic factors may be a stronger determinant of CRF than habitual PA particularly in children (Kemper and Koppes 2006; Ortega et al. 2008a; Martínez-Vizcaíno and Sánchez-López 2008). Furthermore, genetic factors also partly regulate cardiac autonomic nervous system function and may thus partly explain the association between CRF and cardiac autonomic function (Hautala et al. 2009).

### Joint associations of CRF, PA and ST with HRV

We found that boys and girls with a combination of lower PAEE and lower CRF or a combination of higher ST and lower CRF had poorer cardiac autonomic nervous system function than boys and girls with a combination of higher PAEE and higher CRF or a combination of lower ST and higher CRF. Another study showed that adults with higher PA and CRF had higher SDNN and RMSSD than adults with a low PA and CRF (Buchheit and Gindre 2006). However, we observed no differences in RMSSD between boys with higher ST and higher CRF and boys with a combination of lower ST and higher CRF. This suggests that higher CRF may counteract the negative effects of higher ST with cardiac autonomic nervous system function in boys. Furthermore, we found no difference in RMSSD between girls with lower PAEE regardless of the level of CRF. However, RMSSD was higher in girls with higher PAEE and CRF than girls with lower PAEE and lower or higher CRF. These results suggest that PA more beneficially affects cardiac autonomic nervous system function than CRF in girls. The observed differences in the associations of PA, ST, and CRF with cardiac autonomic nervous system function between sexes may be partly explained by differences in maturity, adiposity, insulin, glucose, PA, and CRF between sexes.

### Strength and limitations

The strengths of our study include a relatively large population sample of mainly prepubertal girls and boys with a narrow age range, the objective assessment of PA and ST with combined heart rate and movement sensing, the assessment of CRF by maximal exercise test and using a measure that is not confounded by body size or adiposity, the assessment of cardiac autonomic nervous system function using a wide range of HRV variables, and the opportunity to control for adiposity, other cardiometabolic risk factors, and maturation in the statistical analyses. However, we cannot draw any firm conclusions on causality from the observed relationships of PA, ST, and CRF with cardiac autonomic nervous system function because of the cross-sectional study design.

## Main conclusion

We find higher levels of PA, lower levels of ST, and higher CRF to be associated with better cardiac autonomic function in children. These underline the importance of promoting healthy lifestyle in children. Undoubtedly the dysfunction of the autonomic nervous system plays an important role in cardiovascular health. However, the mechanisms behind that association and how it is modified with lifestyle interventions, especially in the long-term starting from childhood, remains an interesting topic for further studies.

## Electronic supplementary material

Below is the link to the electronic supplementary material.

Supplementary file1 (DOCX 25 kb)

## Abbreviations

BF%: Body fat percentage
CRF: Cardiorespiratory fitness
CRS: Cardiometabolic risk score
HF: High-frequency power
HRV: Heart rate variability
LF: Low-frequency power
LPA: Light physical activity
MET: Metabolic equivalent of task
MPA: Moderate physical activity
MVPA: Moderate-to-vigorous physical activity
PA: Physical activity
PAEE: Physical activity energy expenditure
PANIC: Physical activity and nutrition in children
PHV: Peak height velocity
RMSSD: Root mean square of successive RR interval differences
SDNN: Standard deviation of RR intervals
ST: Sedentary time
VPA: Vigorous physical activity
