# Supplementary material for: Associations of physical activity, sedentary time, and cardiorespiratory fitness with heart rate variability in 6- to 9-year-old children: the PANIC study
Source: Eur J Appl Physiol. 2019 Sep 18;119(11):2487–98. doi: 10.1007/s00421-019-04231-5 (PMC6858383; doi:10.1007/s00421-019-04231-5)
Supplement: Supplementary file 1 — Supplementary file1 (DOCX 25 kb) [file 421_2019_4231_MOESM1_ESM.docx]

Electronic supplementary material

Title: Associations of physical activity, sedentary time, and cardiorespiratory fitness with heart rate variability in 6–9-year-old children: the PANIC Study

Journal name: European Journal of Applied Physiology

Authors:

Aapo Veijalainen^1^, Eero A. Haapala^1,2^, Juuso Väistö^1^, Marja H. Leppänen^2,3^, Niina Lintu^1^, Tuomo Tompuri^1^, Santeri Seppälä^1^, Ulf Ekelund^4^, Mika P. Tarvainen^5,6^, Kate Westgate^7^, Søren Brage^7^, Timo A. Lakka^1,5,8^

^1^ Institute of Biomedicine, School of Medicine, University of Eastern Finland, Kuopio Campus, Finland;

^2^ Faculty of Sport and Health Sciences, University of Jyväskylä, Finland;

^3^ Folkhälsan Research Center, Helsinki, Finland;

^4^ Department of Sport Medicine, Norwegian School of Sport Sciences, Oslo, Norway;

^5^ Department of Clinical Physiology and Nuclear Medicine, Kuopio University Hospital, Kuopio, Finland;

^6^ Department of Applied Physics, University of Eastern Finland, Kuopio, Finland;

^7^ MRC Epidemiology Unit, Institute of Metabolic Science, School of Clinical Medicine, University of Cambridge, Cambridge, UK;

^8^ Kuopio Research Institute of Exercise Medicine, Kuopio, Finland

Corresponding author: Aapo Veijalainen, MD, PhD, Institute of Biomedicine, University of Eastern Finland, Kuopio Campus, PO Box 1627, FI‐70211 Kuopio, Finland. Tel: +358(0)40 748 9029, Fax: +358(0) 17163112, E‐mail: veijalai@uef.fi

| **Online resource 1. Individual associations of sedentary time, physical activity, and cardiorespiratory fitness with mean of RR interval adjusted heart rate variability variables** | | | | | | | | | | |
| --- | --- | --- | --- | --- | --- | --- | --- | --- | --- | --- |
|  | **SDNN** |  | **RMSSD** |  | **LF** |  | **HF** |  | **LF/HF** |  |
|  | **β** | **p** | **β** | **p** | **β** | **p** | **β** | **p** | **β** | **p** |
| **Boys** |  |  |  |  |  |  |  |  |  |  |
| ST | -.050 | .506 | -.082 | .269 | -.026 | .728 | -.038 | .608 | .023 | .752 |
| LPA | .042 | .568 | .033 | .650 | .082 | .276 | .009 | .903 | .075 | .308 |
| MPA | -.069 | .353 | -.012 | .874 | -.119 | .111 | -.050 | .501 | -.060 | .413 |
| MVPA | -.006 | .936 | .056 | .444 | -.084 | .262 | .023 | .758 | -.121 | .100 |
| VPA | .130 | .076 | **.167** | **.022** | .040 | .587 | **.162** | **.026** | **-.176** | **.015** |
| PAEE | .040 | .594 | .096 | .197 | -.027 | .716 | .057 | .446 | -.106 | .152 |
| CRF | .144 | .055 | **.217** | **.003** | .042 | .585 | **.159** | **.033** | **-.170** | **.022** |
| **Girls** |  |  |  |  |  |  |  |  |  |  |
| ST | **-.266** | **<.001** | **-.291** | **<.001** | -.135 | .065 | **-.263** | **<.001** | **.204** | **.005** |
| LPA | **.229** | **.001** | **.244** | **.001** | .139 | .056 | **.204** | **.005** | -.122 | .094 |
| MPA | .083 | .256 | .114 | .118 | -.012 | .874 | .105 | .153 | **-.150** | **.040** |
| MVPA | .132 | .071 | **.164** | **.024** | .000 | 1.000 | **.159** | **.029** | **-.209** | **.004** |
| VPA | **.214** | **.003** | **.235** | **.001** | .035 | .632 | **.244** | **.001** | **-.285** | **<.001** |
| PAEE | **.173** | **.018** | **.206** | **.005** | .022 | .770 | **.193** | **.008** | **-.232** | **.001** |
| CRF | -.005 | .946 | .062 | .403 | -.104 | .160 | .054 | .467 | **-.182** | **.014** |
| Values are standardized regression coefficients (β) and P‐values from linear regression analyses in which each ST, PA, and CRF variable was entered individually with years from peak height velocity into the models. P-values <0.05 indicating statistically significant associations are in bold. Abbreviations: SDNN, standard deviation of all RR intervals; RMSSD, root mean square of successive RR interval differences; LF, low frequency power; HF, high frequency power; ST, sedentary time; PA, physical activity; LPA, light PA; MPA, moderate PA; MVPA, moderate-to-vigorous PA; VPA, vigorous PA; PAEE, physical activity energy expenditure; CRF, cardiorespiratory fitness | | | | | | | | | | |

| **Online resource 2. Mutually adjusted associations of sedentary time, physical activity, and cardiorespiratory fitness with mean of RR interval adjusted heart rate variability variables** | | | | | | | | | | |
| --- | --- | --- | --- | --- | --- | --- | --- | --- | --- | --- |
|  | **SDNN** |  | **RMSSD** |  | **LF** |  | **HF** |  | **LF/HF** |  |
|  | **β** | **p** | **β** | **p** | **β** | **p** | **β** | **p** | **β** | **p** |
| **Boys** |  |  |  |  |  |  |  |  |  |  |
| ST | -.065 | .613 | -.032 | .796 | -.157 | .229 | .023 | .858 | -.200 | .109 |
| PAEE | -.088 | .522 | -.047 | .728 | -.164 | .241 | -.036 | .789 | -.127 | .341 |
| CRF | .149 | .069 | **.220** | **.007** | .053 | .519 | .160 | .050 | **-.160** | **.045** |
| **Girls** |  |  |  |  |  |  |  |  |  |  |
| ST | **-.372** | **.004** | **-.375** | **.004** | **-.366** | **.005** | **-.321** | **.013** | .035 | .787 |
| PAEE | -.087 | .505 | -.091 | .486 | -.189 | .155 | -.055 | .676 | -.129 | .327 |
| CRF | -.012 | .880 | .055 | .487 | -.076 | .348 | .042 | .602 | -.136 | .090 |
| Values are standardized regression coefficients (β) and P‐values from linear regression analyses in which ST, PAEE, and CRF were entered simultaneously with years from peak height velocity, body fat percentage, and cardiometabolic risk score into the models. P-values <0.05 indicating statistically significant associations are in bold. Abbreviations: ST, sedentary time; PA, physical activity; CRF, cardiorespiratory fitness; SDNN, standard deviation of all RR intervals; RMSSD, root mean square of successive RR interval differences; LF, low frequency power; HF, high frequency power; PAEE, PA energy expenditure. | | | | | | | | | | |
